# Integrating single-cell RNA-seq and machine learning to dissect polyamine metabolism in metabolic dysfunction-associated steatotic liver disease

**DOI:** 10.3389/fmed.2026.1786869

**Published:** 2026-04-29

**Authors:** Peng Zou, Lin Sun, Zhibin Lin

**Affiliations:** Department of Hepatobiliary Surgery, Xijing Hospital, Fourth Military Medical University, Xi’an, China

**Keywords:** machine learning, macrophage, metabolic dysfunction-associated steatotic liver disease, polyamine metabolism, single-cell RNA sequencing

## Abstract

**Background:**

Dysregulated immunometabolism is central to the pathogenesis of metabolic dysfunction-associated steatotic liver disease (MASLD). Although polyamines contribute to cellular stress responses and immune-cell function, their cell-type-specific transcriptional associations within the hepatic immune microenvironment remain incompletely understood.

**Methods:**

We assessed polyamine metabolism in MASLD at the single-cell level using AUCell, UCell, singscore, and AddModuleScore. To find metabolism-related genes, we performed differential analyses. We then combined six machine learning methods—including LASSO, Random Forest, XGBoost, GBM, SVM, and Boruta—to identify and sort robust disease genes. We further evaluated these findings using bulk transcriptomic datasets and a CDAA-induced MASH mouse model.

**Results:**

We observed notable differences in polyamine metabolic activity among various liver cell types, with relatively higher levels detected in macrophages, cholangiocytes, and stromal cells. PSMB7 and PSMD7 emerged as proteasome-associated candidate genes that were enriched in macrophages and upregulated in MASLD. Higher expression of these genes was associated with immune-related transcriptional programs, including antigen-processing/presentation signatures and predicted intercellular communication pathways involving MIF- and TNFSF13B-related signaling. Their upregulation was further supported by bulk RNA analyses and the CDAA-induced MASH model.

**Conclusion:**

Our single-cell analysis showed clear heterogeneity in polyamine-metabolism-related states in MASLD, with macrophages emerging as a major associated cell population. PSMB7 and PSMD7 emerged as proteasome-associated candidate markers enriched in macrophage populations with elevated polyamine-metabolism scores, providing a framework for future mechanistic investigation.

## Graphical abstract

**Figure:**
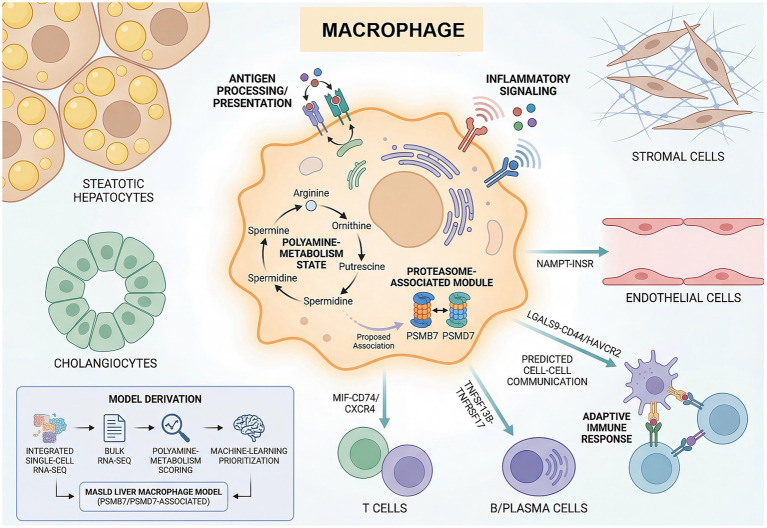

## Introduction

Metabolic dysfunction-associated steatotic liver disease (MASLD), formerly known as nonalcoholic fatty liver disease (NAFLD), has emerged as the leading cause of chronic liver disease globally amid rising obesity and type 2 diabetes prevalence (1–3). MASLD is not a single disease but a continuum spanning from simple hepatic steatosis to metabolic dysfunction-associated steatohepatitis (MASH) with varying degrees of fibrosis (4). MASH, characterized by lobular inflammation and hepatocyte injury, is a major driver of liver fibrosis and hepatocellular carcinoma, severely impacting patients’ quality of life and prognosis (4, 5). The pathogenesis of MASLD is complex, with immune cells playing a central role (6, 7). Notably, as a metabolic disorder, MASLD significantly remodels the metabolic patterns of hepatic immune cells. This “metabolic reprogramming” of immune cells is considered a key mechanism driving progression from simple steatosis to MASH (8).

Hepatic macrophages are key drivers of inflammation and fibrosis in this altered environment. This population includes resident Kupffer cells (KCs) and recruited monocyte-derived macrophages (MoMFs). During MASH, metabolic stress signals like lipotoxicity cause these cells to change their phenotype. Once activated, they release cytokines and chemokines that recruit other immune cells and activate hepatic stellate cells, maintaining liver injury. Therefore, understanding the metabolic needs of these macrophages is essential for finding new therapeutic targets.

Polyamine metabolism is a critical branch of arginine metabolism, not only sustaining the homeostasis of cell proliferation and autophagy but also serving as a core component of immunometabolism (9, 10). Although polyamine homeostasis disruption is known to drive oxidative stress and fibrosis (11), its regulatory mechanisms within the immune microenvironment remain unexplored in the complex pathological setting of MASH.

In our study, we analyzed polyamine metabolism in MASLD using both single-cell and bulk RNA sequencing. Through this research, we found that two genes involved in the proteasome, PSMB7 and PSMD7, were repeatedly associated with metabolic changes observed in MASLD. We further examined the association of these genes with immune-related transcriptional features and evaluated their expression in a CDAA-induced MASH mouse model. Together, these analyses provide a hypothesis-generating framework for understanding how macrophage-centered metabolic remodeling may relate to immune dysregulation during MASLD progression.

## Methods

### Data acquisition and processing

All data used in this study were collected from the Gene Expression Omnibus (GEO) database.^1^ Three transcriptomic cohorts of metabolic dysfunction-associated steatohepatitis (MASLD) liver tissue—GSE48452, GSE89632, and GSE66676—were selected from GEO for bulk-level expression analysis. At the same time, the single-cell transcriptomics dataset GSE136103 was obtained for resolving the cellular composition and transcriptional characteristics of MASLD liver tissue at single-cell resolution (12). Sources for each dataset are shown in Supplementary Table 1. To study abnormalities in polyamine metabolism in MASLD, gene sets related to polyamine metabolism were compiled from the MSigDB resource in GSEA for subsequent analysis (Supplementary Table 2). The single-cell dataset GSE136103 was processed with the Seurat R package (13). A Seurat object was generated first, and quality control was performed at the cellular level: cells with 200 to 7,000 detected genes and mitochondrial gene content below 30% were kept. After this step, 40,603 high-quality cells remained for further analysis. The data were then normalized by LogNormalize, highly variable genes were selected, principal component analysis (PCA) and UMAP dimensionality reduction were carried out, and cell clustering was done using the clustering function in Seurat. Marker genes for each cell cluster were identified through differential expression, and cell type annotation was based on integrating commonly reported marker genes from the literature. For bulk cohorts GSE48452, GSE89632, and GSE66676, data preprocessing followed the standard workflows of each platform, including background correction, normalization, and log2 transformation as needed.

### Polyamine metabolism scoring algorithm in scRNA-seq data

To assess polyamine metabolic activity at the single-cell level, we combined four different analytical methods: AUCell (14), UCell (15), singscore (16) and AddModuleScore (17). AUCell allowed us to measure gene set enrichment by calculating the area under the curve (AUC) for the metabolic gene signature among the top-expressed genes in each cell. In parallel, UCell provided scores for the gene signature based on the Mann–Whitney U statistic. Singscore examined the concordance of the gene set by calculating the normalized average rank relative to expected extremes. AddModuleScore further contributed by comparing the average expression of the target gene set to that of random control genes. By bringing together the results from these various methods, we arrived at a comprehensive score reflecting the polyamine metabolic activity in each cell, which strengthened the reliability of our analysis.

### Differential gene expression and functional enrichment analysis

Based on the scoring results, cells were divided into two groups—high and low polyamine metabolic activity group. To find differentially expressed genes (DEGs) between these groups, the FindMarkers function in the Seurat package was employed. DEGs were defined as those with an | log2 fold change | > 0.25 and an adjusted *p*-value < 0.05. At the same time, a correlation analysis was conducted to identify genes most strongly associated with polyamine metabolic activity, and the top candidates—for example, the top 30—were chosen for further analysis. Genes appearing in both the differential analysis and the correlation analysis were considered to be important shared genes involved in the upregulation of polyamine metabolism. These key genes were then analyzed with Gene Ontology (GO) and KEGG pathway enrichment tools, such as clusterProfiler in R, to help interpret their biological functions and the pathways they are connected to.

### Machine learning screening of key genes in polyamine metabolism in MASLD

To identify robust candidate genes associated with polyamine-metabolism-associated transcriptional states in MASLD, we applied six machine-learning algorithms for feature selection, including Boruta (18), LASSO (19), Random Forest (RF) (20), eXtreme Gradient Boosting (XGBoost) (21), Gradient Boosting Machine (GBM) (22), and Support Vector Machine (SVM) (23). The idea behind this ensemble approach was to leverage each method’s strengths in identifying key features and modeling complex relationships, and, at the same time, minimize bias that could arise from depending on one single algorithm. With Boruta, we compared real gene features to randomly shuffled “shadow” features, enabling us to confidently distinguish meaningful genes from noise. For LASSO regression, we utilized the glmnet package and applied L1 regularization along with 10-fold cross-validation, which helped eliminate genes that did not contribute much. Random Forest allowed us to rank genes based on their role in enhancing prediction accuracy. Both GBM and XGBoost are gradient boosting methods that refined the model by correcting errors from previous rounds—XGBoost also included early stopping to prevent overfitting. Finally, we selected SVM to capture more complicated non-linear relationships between gene expression and disease status, and we improved gene selection through recursive feature elimination.

### GSVA enrichment analysis

We performed gene set variation analysis (GSVA) using the “GSVA” package in R to identify pathway differences between different groups, aiming to explore potential differential biological mechanisms. Gene sets were sourced from the MSigDB database.^2^ Multiple testing was corrected using the Benjamini–Hochberg method, with FDR < 0.05 indicating significant enrichment, thereby identifying key signaling pathways. Additionally, we performed PPI and miRNA network analyses to assess underlying regulatory relationships among genes.^3^

### Immune infiltration analysis

CIBERSORT is a deconvolution algorithm based on support vector regression that utilizes global gene expression profiles to infer the immune cell composition within complex tissues (24). In this study, we applied the CIBERSORT algorithm to analyze samples from the MASLD group and control group, estimating the relative proportions of 22 infiltrating immune cell types within each sample, with the sum of all immune cell proportions constrained to 1. To ensure the reliability of the deconvolution results, only samples with CIBERSORT results *p* < 0.05 were retained for subsequent analysis. Subsequently, we compared the immune cell infiltration profiles between MASLD and control tissues to identify differentially infiltrating immune cell types. Concurrently, Spearman correlation analysis was performed to assess the relationship between polyamine metabolism-related gene expression levels and the proportion of various immune cell types, with *p* < 0.05 considered statistically significant.

### Cell communication

We employed CellChat to analyze gene expression data and explore potential cell–cell communication networks (25). Following CellChat’s standard analysis workflow, the default CellChatDB was used as the ligand–receptor interaction database. Cell type-specific interactions were inferred by identifying ligands or receptors highly expressed in specific cell populations. Enhanced interactions within ligand–receptor signaling pathways were determined when either ligand or receptor showed significantly elevated expression. This analysis helped characterize variations in cell communication patterns across different cell types.

### Pseudo-time analysis

Pseudotime and trajectory analysis of cells based on single-cell transcriptomic expression profiles to reconstruct developmental/state evolution processes. First, the gene–cell expression matrix is extracted from the Seurat object as input data, and Monocle 2 software is used for reverse-chronological analysis (26, 27). Within this analytical framework, Monocle performs dimensionality reduction and clustering based on gene expression patterns, categorizing cells into distinct developmental/functional states. By identifying genes dynamically changing along trajectories, it arranges individual cells on a continuous pseudotime axis, thereby constructing underlying developmental trajectories and branching structures.

### Mouse MASH model

Male C57BL/6 J mice (8 weeks old) were purchased from Beijing Vital River Laboratory Animal Technology Co., Ltd. A choline-deficient, L-amino acid-defined (CDAA) diet (Research Diet, A06071209, United States) for 10 weeks are fed to establish a mouse model of metabolic dysfunction-associated steatohepatitis (28). Mice in the control group were fed a normal control diet (Xietong Bio-engineering, XTI01WC, China). All animals were maintained under specific pathogen-free conditions with a 12-h light/dark cycle and had ad libitum access to food and water. All experimental procedures were conducted in compliance with the National Academies’ Guide for the Care and Use of Laboratory Animals and were approved by the Animal Ethics Committee of Fourth Military Medical University (Xi’an, China).

### qRT-PCR

Following the reagent manual, total RNA was extracted from mouse liver tissue using Trizol Reagent (AG21101, Accurate Biology, China), and cDNA was synthesized via reverse transcription using Evo M-MLV RT Master Mix (AG11706, Accurate Biology, China). Subsequently, qPCR was performed on a real-time fluorescent quantitative PCR instrument (12,011,319, BIORAD, United States). The reaction system had a total volume of 10 μL, containing cDNA, SYBR Green Pro Taq HS Premix (AG11701, Accurate Biology, China), and specific primers. ACTIN (NM_007393.5, F: GGCTGTATTCCCCTCCATCG R: CCAGTTGGTAACAATGCCATGT) served as the internal reference gene, and the expression levels of target genes (PSMB7, NM_011187.1, F: GTGTCGGTGTTTCAGCCAC, R: GTGCCAGTTTTCCGAGCT, TTC; PSMD7, NM_010817, F: GGTGGATCATTTCAACCGAATTG, R: AAGGTACTGCAAAACTGTTGGAT) were expressed as fold-change relative to the control group.

### Western blot

Processed tissues were lysed on ice in RIPA buffer supplemented with protease inhibitors (P1045, Beyotime, China). The lysates were then centrifuged at 12,000 × g for 10 min at 4 °C, and the supernatants were collected. Total protein concentration was measured using a BCA Protein Assay Kit (23,227, Thermo Fisher Scientific, United States). Equal amounts of protein (20 μg per lane) were separated on 10% SDS-PAGE gels at 120 V for 2 h and subsequently transferred onto PVDF membranes (IPFL00010, Millipore, United States). Membranes were blocked with 5% nonfat milk in TBST for 1 h at room temperature and then incubated overnight at 4 °C with gentle agitation with the following primary antibodies: anti-PSMB7 (1:1000, GB113453-50, Servicebio, China), anti-PSMD7 (1:1000, A5356, ABclonal, China), and anti-*β*-actin (1:5000, GB15003, Servicebio, China) as the loading control. After three washes with TBST, membranes were incubated with the appropriate HRP-conjugated secondary antibody (1:5000, 511,203, Zenbio, China) for 1 h at room temperature. Protein bands were visualized and quantified using Image Lab software. Relative protein expression was calculated as the band intensity of the target protein normalized to that of *β*-actin.

### Data analysis

Data processing, statistical analysis, and visualization for this study were performed using R software (version 4.3.3) and GraphPad Prism software (version 9.5, GraphPad Software, United States). Differences in continuous variables between two groups were assessed using Student’s t-test or Wilcoxon signed-rank test, selected based on data distribution characteristics. Comparisons among multiple groups were performed using one-way analysis of variance (ANOVA). Categorical variables were analyzed using chi-square tests or Fisher’s exact tests. Correlations between variables were assessed via Pearson correlation analysis. Where applicable, multiple comparisons were corrected using the false discovery rate (FDR) method. All *p*-values were calculated for two-tailed tests, with *p* < 0.05 considered statistically significant.

## Results

### The scRNA-seq profiling of MASLD

**Figure 1:**
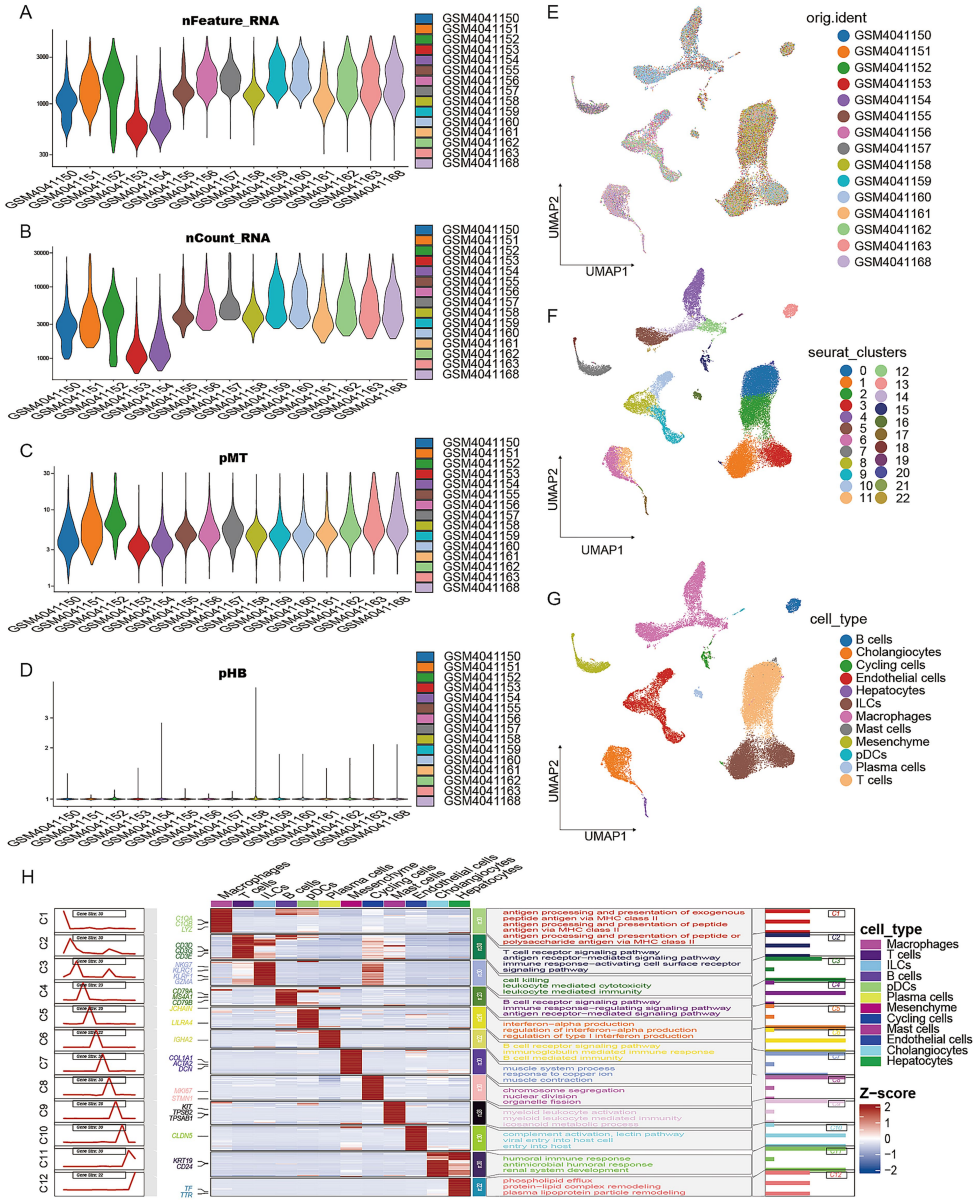
The scRNA-seq profiling of MASLD (metabolic dysfunction-associated steatotic liver disease). **(A–D)** Quality control for scRNA data. **(E)** Batch effects between samples. **(F)** Seurat clusters in UMAP (Uniform Manifold Approximation and Projection) plot. **(G)** Cell annotations show 12 cell types. **(H)** The relevant pathways enriched by GO (gene ontology) analysis and marker genes of 12 cell types mentioned above.

Prior to subsequent analysis, all samples underwent rigorous quality control (Figures 1A–D), ultimately yielding 15 samples (11 normal controls and 4 abnormal cases) for single-cell RNA sequencing analysis. Principal component analysis revealed stable overall cell distribution across all samples with low sensitivity to batch effects (Figure 1E). Following UMAP nonlinear dimensionality reduction analysis, 23 distinct cell subpopulations were identified (Figure 1F). Based on expression profiles of classical marker genes (e.g., CD3D, CD79A, FCGR3A), we successfully annotated 12 major cell types, including T cells, B cells, ILCs, macrophages, plasma cell-like dendritic cells, mesenchymal cells, hepatocytes, cholangiocytes, cycling cells, endothelial cells, mast cells, and plasma cells (Figure 1G). Pathway enrichment analysis of these 12 cell types provides valuable insights for better understanding their functions (Figure 1H).

### Polyamine metabolism in MASLD

**Figure 2:**
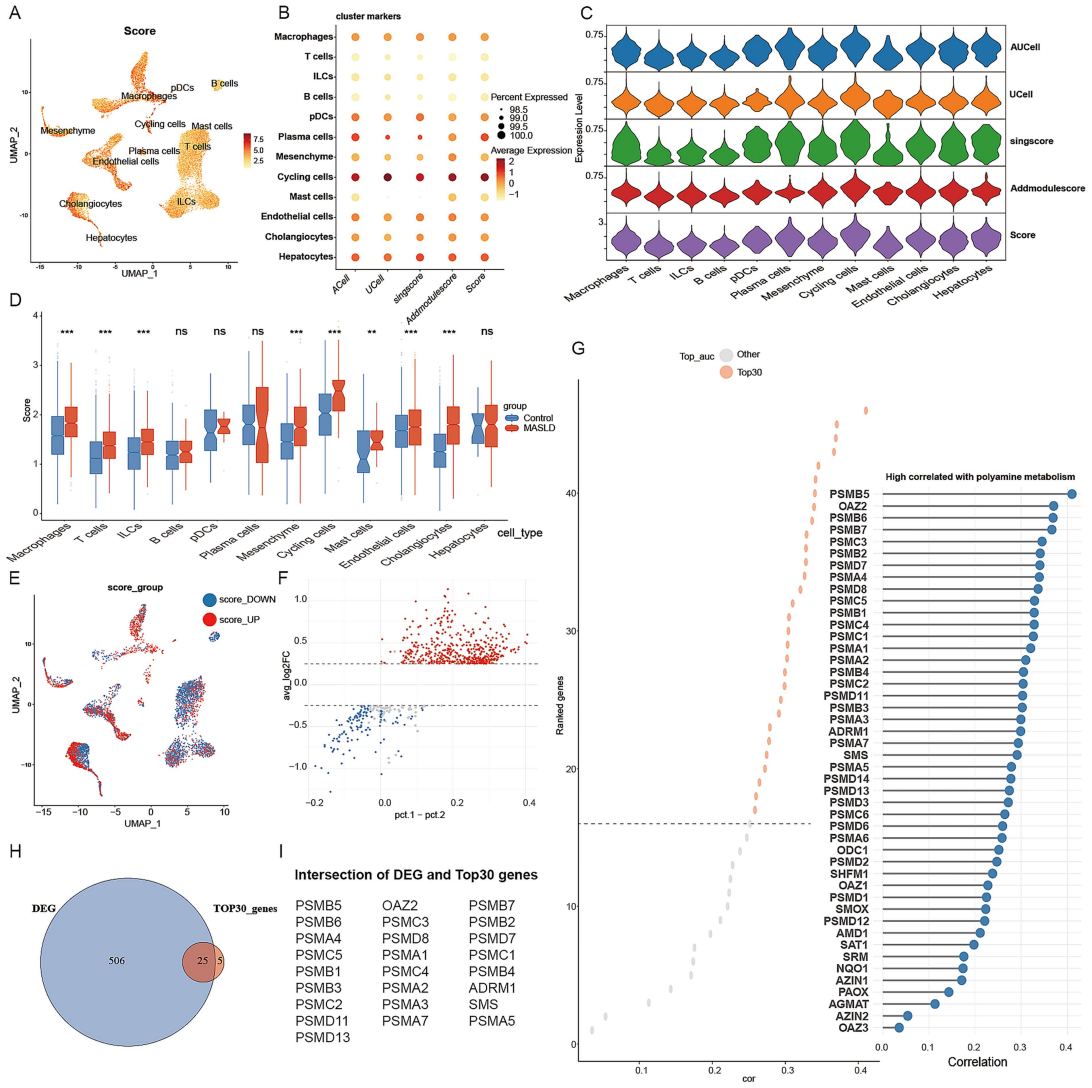
Analysis of polyamine metabolism in MASLD. **(A–C)** Polyamines metabolism activity of each cell types using AUCell, UCell, singscore and Addmodulescore algorithms. **(D)** Violin plot shows the difference between control and MASLD in each cell type. **(E)** All cells are divided into two groups based on polyamine metabolic activity: the high activity group (red) and the low activity group (blue). **(F)** Differential analysis between the high and low polyamine metabolic activity groups. **(G)** Correlation analysis between Scoring expression and gene related to polyamine metabolism. **(H,I)** The up-regulated genes most associated polyamine metabolism.

To systematically evaluate polyamine metabolism activity across different cell types, we scored polyamine metabolism-related genes using four algorithms—AUCell, UCell, singscore, and AddModuleScore—based on single-cell transcriptomic data (Figures 2B,C). The results from these methods showed concordant results, indicating substantial heterogeneity in polyamine metabolic activity across different cell types (Figure 2A). Specifically, relatively higher scores were observed in macrophages, mesenchymal cells, cholangiocytes, hepatocytes, endothelial cells, and plasma cells, whereas lower scores were observed in B cells, T cells, mast cells, and ILCs (Figures 2B,C).

Further comparison between the MASLD group and healthy controls revealed marked remodeling of polyamine metabolic activity across multiple cell types (Figure 2D). These scores were significantly elevated in macrophages, cholangiocytes, and stromal cells within the MASLD group (*p* < 0.05). UMAP clustering indicated that cells with high metabolic activity predominantly clustered within macrophages, cholangiocytes, and stromal cells (Figure 2A).

**Figure 3:**
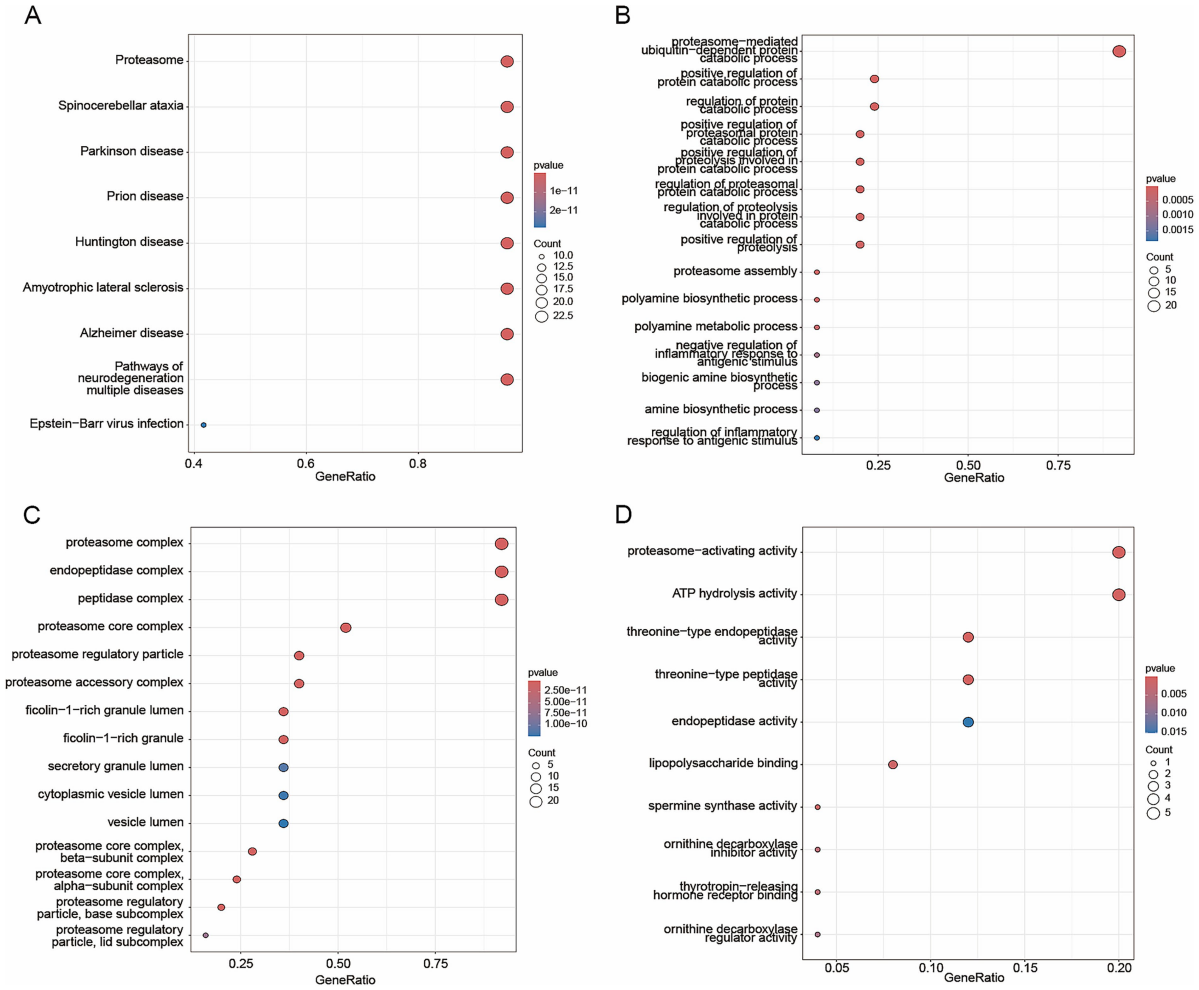
KEGG and GO enrichment analysis of overlapping genes. **(A)** KEGG enrichment analysis. **(B–D)** GO enrichment analysis.

Based on integrated scoring, we divided all 40,603 cells into high- and low-score groups by the median value. We found that the high-score group primarily comprised macrophages, endothelial cells, and cholangiocytes (Figure 2E). Differential analysis between the high- and low-score groups identified 531 genes significantly upregulated in the high-expression group (Figure 2F). In parallel, polyamine-metabolism-related gene set was retrieved from MSigDB, and the top 30 genes ranked by correlation with the integrated score were selected for overlap analysis (Figure 2G). Intersecting these differentially expressed genes with polyamine metabolism-related genes yielded 25 core upregulated genes most closely associated with polyamine metabolic activity (Figures 2H,I). KEGG/GO enrichment analysis of these core upregulated genes strongly focused on the proteasome system, protein degradation regulation, and polyamine biosynthesis pathways (Figures 3A–D).

### Screening of the optimal genes by machine learning

**Figure 4:**
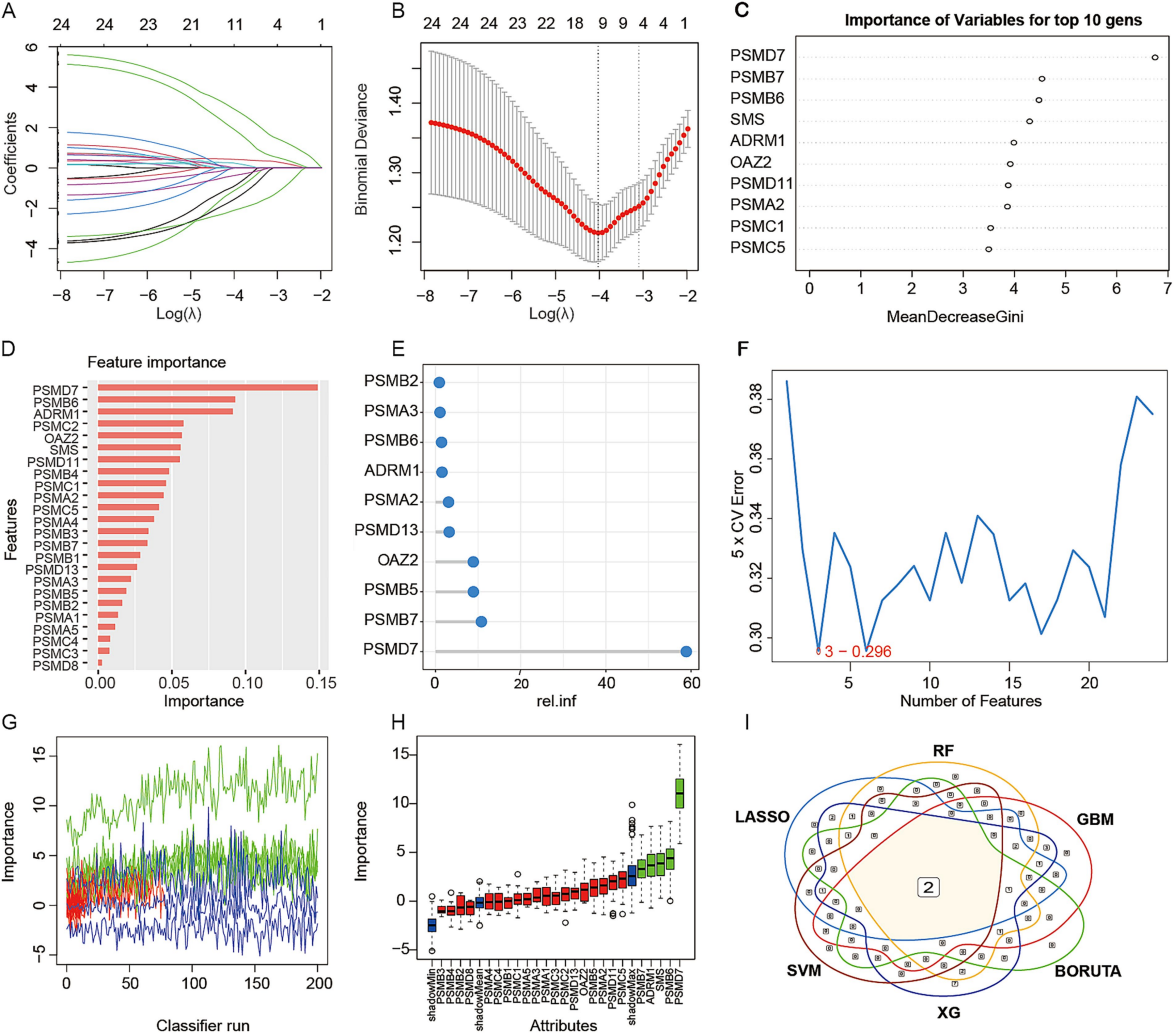
Identification of key marker genes using machine learning. **(A,B)** The LASSO (Least Absolute Shrinkage and Selection Operator) algorithm. **(C)** The random forest algorithm. **(D)** The XGBoost algorithm. **(E)** GBM (Gradient Boosting Machine) algorithm. **(F)** SVM (Support Vector Machine) algorithm. **(G,H)** Boruta algorithm. **(I)** Venn diagram shows the two optimal key genes shared among the six machine learning results mentioned above.

**Figure 5:**
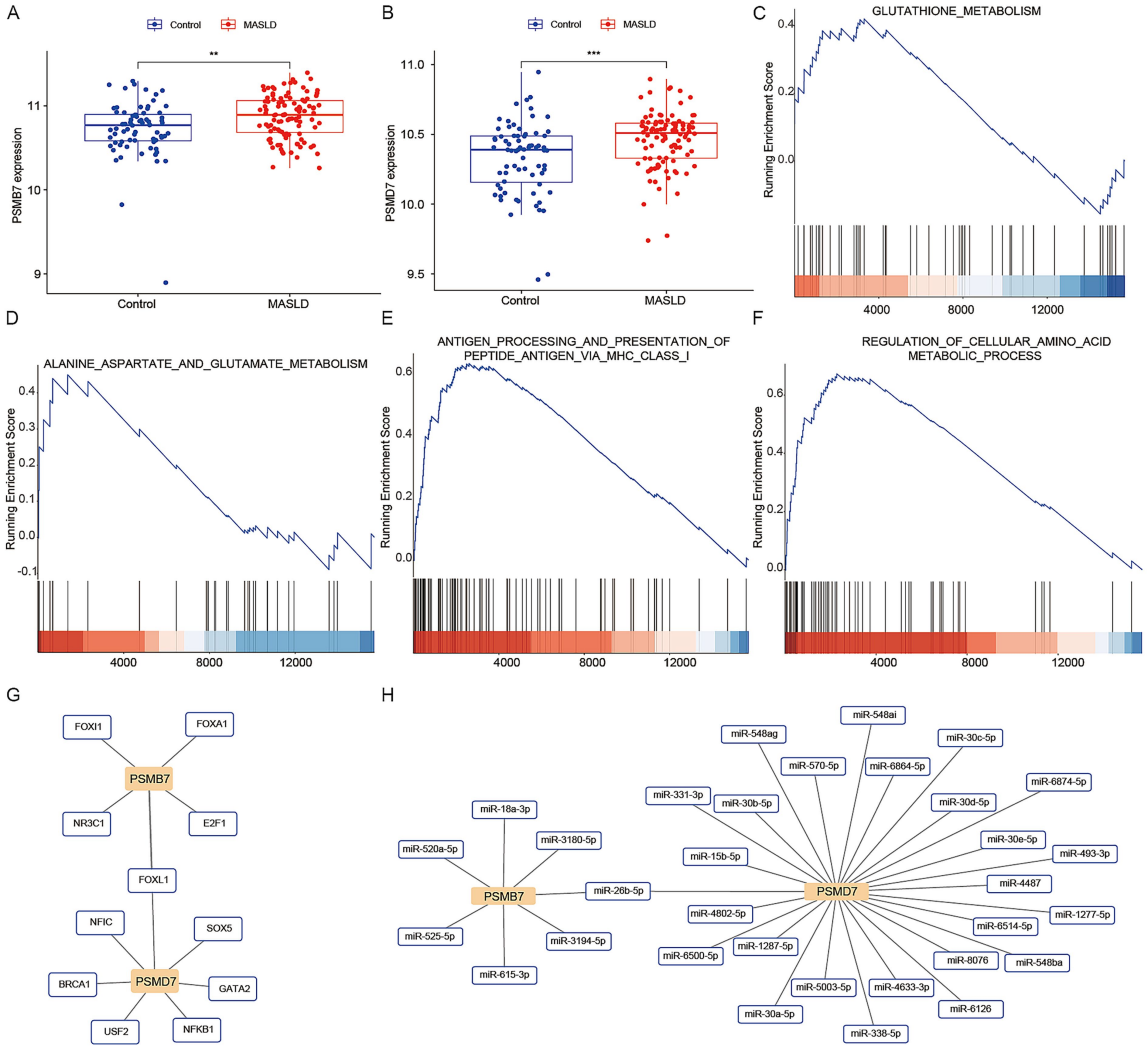
The expression levels, enrichment analysis, and regulatory network prediction of PSMB7 and PSMD7. **(A,B)** Bulk RNA-seq analysis showing upregulation of PSMB7 and PSMD7 in MASLD. **(C–F)** The ssGSEA enrichment analysis of PSMB7 and PSMD7. **(G,H)** Predicted transcription factor and miRNA regulatory networks centered on PSMB7 and PSMD7.

We applied six machine learning algorithms—LASSO (Figures 4A,B), Random Forest (Figure 4C), XGboost (Figure 4D), GBM (Figure 4E), SVM (Figure 4F), and Boruta (Figures 4G,H)—to screen for candidate feature genes in the training set. Intersection of genes jointly selected by these algorithms identified two robust candidates, PSMD7 and PSMB7 (Figure 4I). Bulk transcriptomic analysis showed that both genes were upregulated in MASLD (Figures 5A,B). ssGSEA performed in samples stratified by PSMB7 or PSMD7 expression revealed enrichment of amino-acid-metabolism-related pathways, including glutathione and alanine metabolism, as well as immune-related pathways such as antigen-processing/presentation signatures (Figures 5C–F). Notably, both PSMB7- and PSMD7-high expression groups also enriched immune-related pathways, such as antigen presentation-related pathways, suggesting that these two proteins may jointly participate in regulating the immune microenvironment while undergoing metabolic reprogramming. We then constructed predicted transcription factor and miRNA regulatory networks centered on these genes. Results revealed that both PSMB7 and PSMD7 form close interactions with multiple transcription factors. PSMB7 primarily connects with factors like FOXA1, FOXI1, E2F1, and NR3C1, while PSMD7 forms regulatory modules with FOXL1, SOX5, GATA2, NFKB1, BRCA1, USF2, and NFIC (Figure 5G), suggesting that both may integrate multiple upstream signaling pathways to exert their combined effects. Concurrently, miRNA network analysis revealed that PSMB7 is targeted by multiple miRNAs (e.g., miR-18a-3p, miR-520a-5p, miR-525-5p, miR-615-3p, miR-3180-5p, miR-3194-5p, miR-26b-5p), while PSMD7 undergoes broader miRNA regulation (including miR-30b-5p, miR-30e-5p, miR-331-3p, miR-1287-5p, miR-5003-5p, miR-4633-3p, miR-570-5p, and multiple miR-548 family members) (Figure 5H). These analyses positioned PSMB7 and PSMD7 within broader transcriptional and post-transcriptional regulatory contexts and further supported their prioritization as candidate genes associated with MASLD-related metabolic and immune features.

### Association of PSMB7 and PSMD7 with immune-cell composition and immune-related pathway alterations

**Figure 6:**
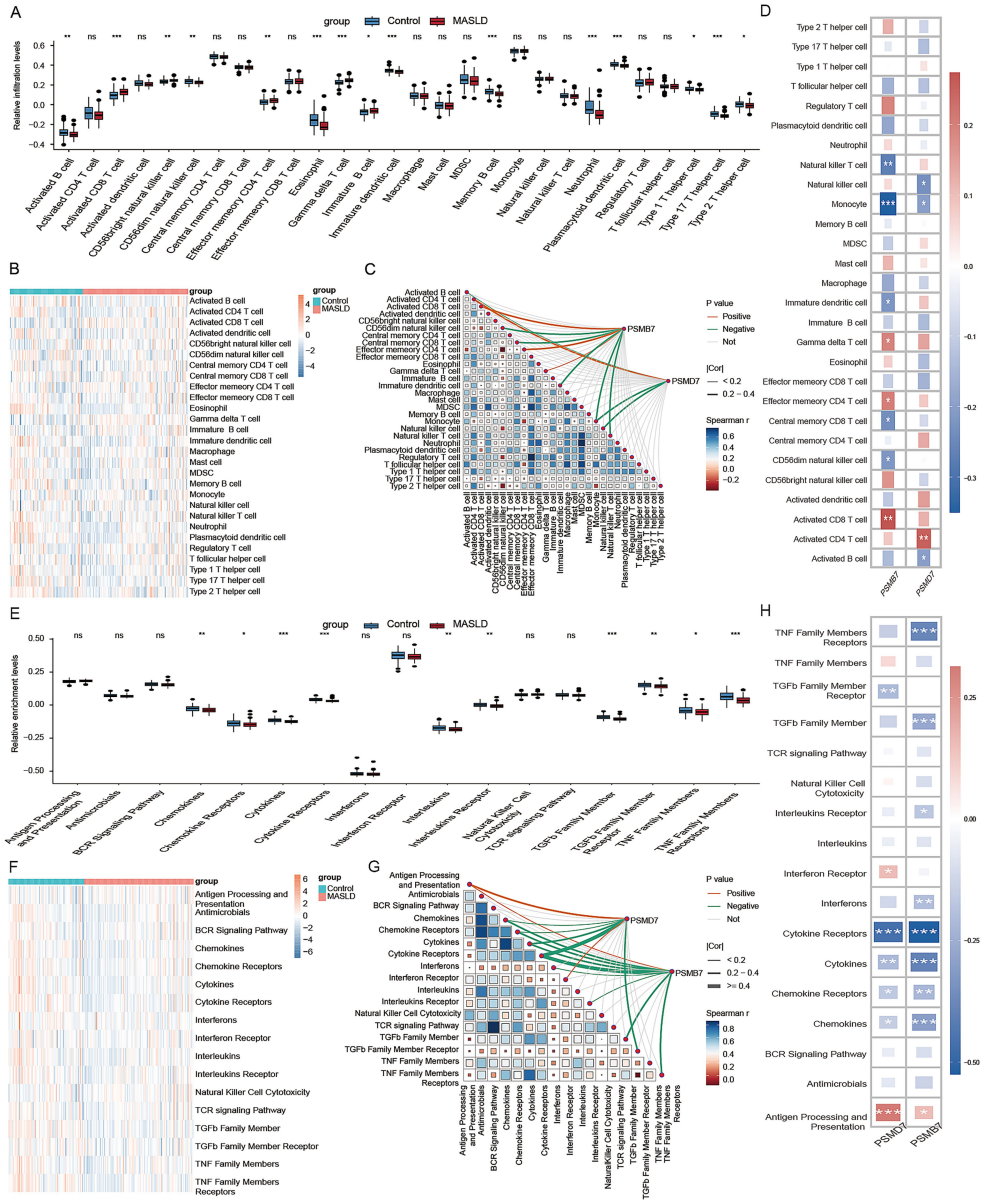
Associations of PSMB7 and PSMD7 with immune-cell composition and immune-related pathway alterations. **(A,B)** Analysis of changes in immune cell subsets between MASLD and control groups using CIBERSORT. **(C,D)** Correlation analysis of PSMB7 and PSMD7 with immune cell infiltration. **(E,F)** Analysis of immune-related signaling pathways in the MASLD and control groups using CIBERSORT. **(G,H)** Correlation analysis between PSMB7/PSMD7 expression and immune-related pathways.

We next evaluated associations between PSMB7/PSMD7 expression and estimated immune-cell composition as well as immune-related pathways (Figure 6). Compared with the control group, MASLD showed significantly abnormal infiltration of multiple innate and adaptive immune cell types (Figures 6A,B). PSMB7 and PSMD7 expression was positively correlated with activated CD8 + T cells, effector memory CD4 + T cells, activated CD4 + T cells, and γδ T cells, and negatively correlated with immature dendritic cells (Figures 6C,D). This suggests that higher PSMB7/PSMD7 expression is associated with altered immune-cell composition in MASLD. Analysis of immune-related pathways further showed broad transcriptomic alterations in cytokine, chemokine, interferon, and TNF family-related signaling (Figures 6E,F). PSMB7 and PSMD7 exhibited similar correlation patterns across these pathways, including positive associations with antigen-processing/presentation and interferon-receptor-related signatures (Figures 6G,H). Together, these findings support an association between PSMB7/PSMD7 expression and immune-related transcriptomic features in MASLD.

### Validation of PSMB7 and PSMD7 in scRNA-seq data

**Figure 7:**
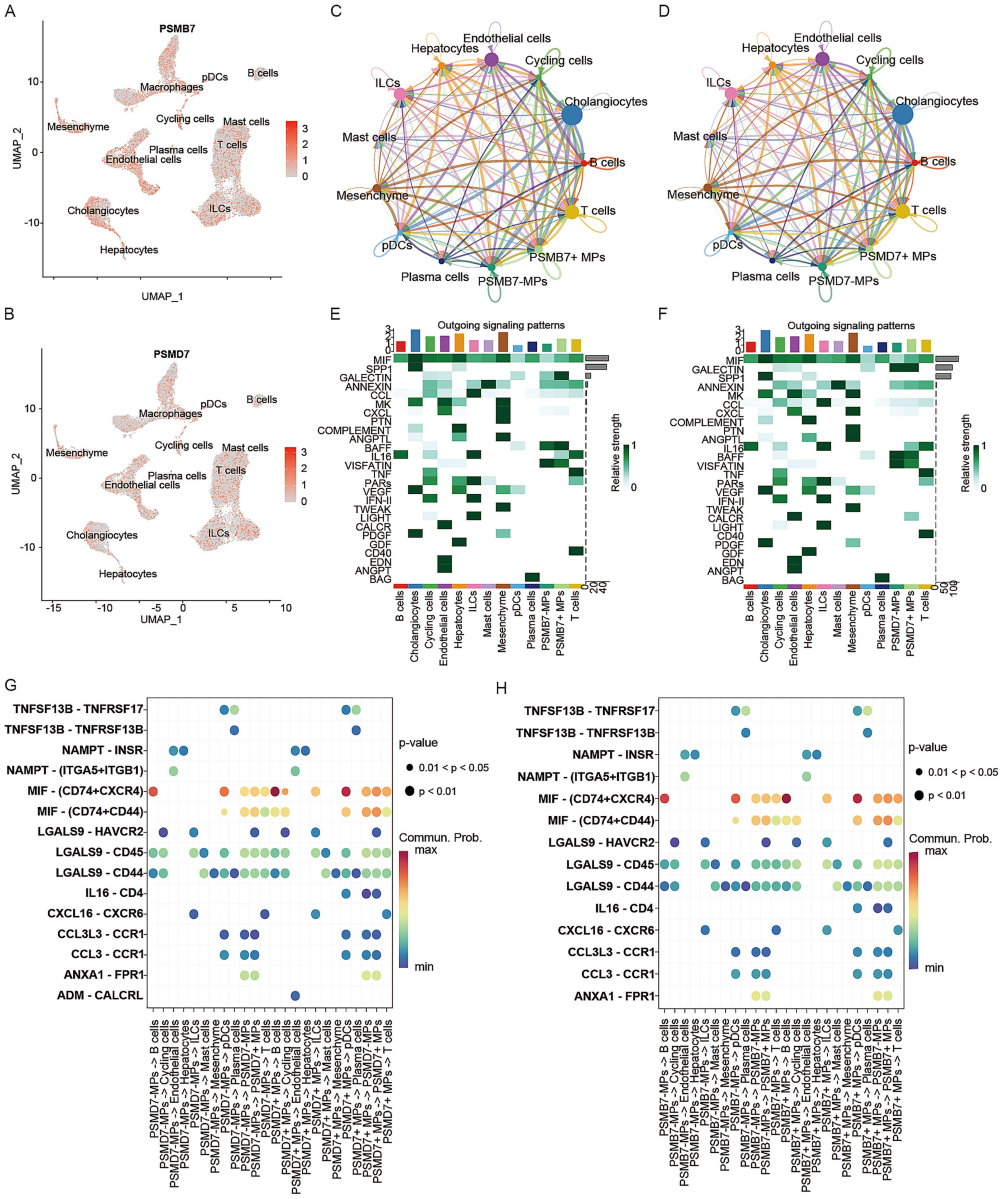
Validation of PSMB7 and PSMD7 in scRNA-seq. **(A,B)** Expression of PSMB7 and PSMD7 across different cell types. **(C,D)** Predicted communication number between PSMB7+/PSMD7 + macrophages and other cell types. **(E,F)** Signaling patterns between PSMB7 + Macrophages and PSMD7 + Macrophages and other cell types. **(G,H)** Predicted ligand-receptor interactions between PSMB7+/PSMD7 + macrophages and other cell populations.

At single-cell resolution, we characterized the cellular localization of PSMB7 and PSMD7 within the hepatic microenvironment (Figures 7A,B). UMAP expression profiles showed baseline expression in hepatocytes and cholangiocytes but relatively higher enrichment in immune-cell populations, particularly macrophages, plasma cells, and plasmacytoid dendritic cells. We therefore focused on macrophage subpopulations with high expression of these genes (defined as PSMB7 + MPs and PSMD7 + MPs) and evaluated their predicted communication networks using CellChat. Overall network analysis revealed that PSMB7 + and PSMD7 + MPs occupy relatively central positions in the hepatic cell interaction network, and display dense predicted signaling links with hepatocytes, endothelial cells, and lymphoid cells (Figures 7C,D). Analysis of outgoing signaling patterns further suggested that these macrophage subpopulations may act as important senders of MIF-, GALECTIN-, ANNEXIN-, and BAFF-related signals while also receiving some inputs from the microenvironment (Figures 7E,F). Candidate ligand-receptor analysis highlighted recurrent interaction axes involving MIF-CD74/CXCR4, LGALS9-CD44/HAVCR2, TNFSF13B-TNFRSF17, and NAMPT-INSR (Figures 7G,H). These findings suggest that macrophages with high PSMB7/PSMD7 expression are associated with altered immune communication patterns in MASLD, particularly in predicted interactions with T cells, B cells, endothelial cells, and plasma cells.

**Figure 8:**
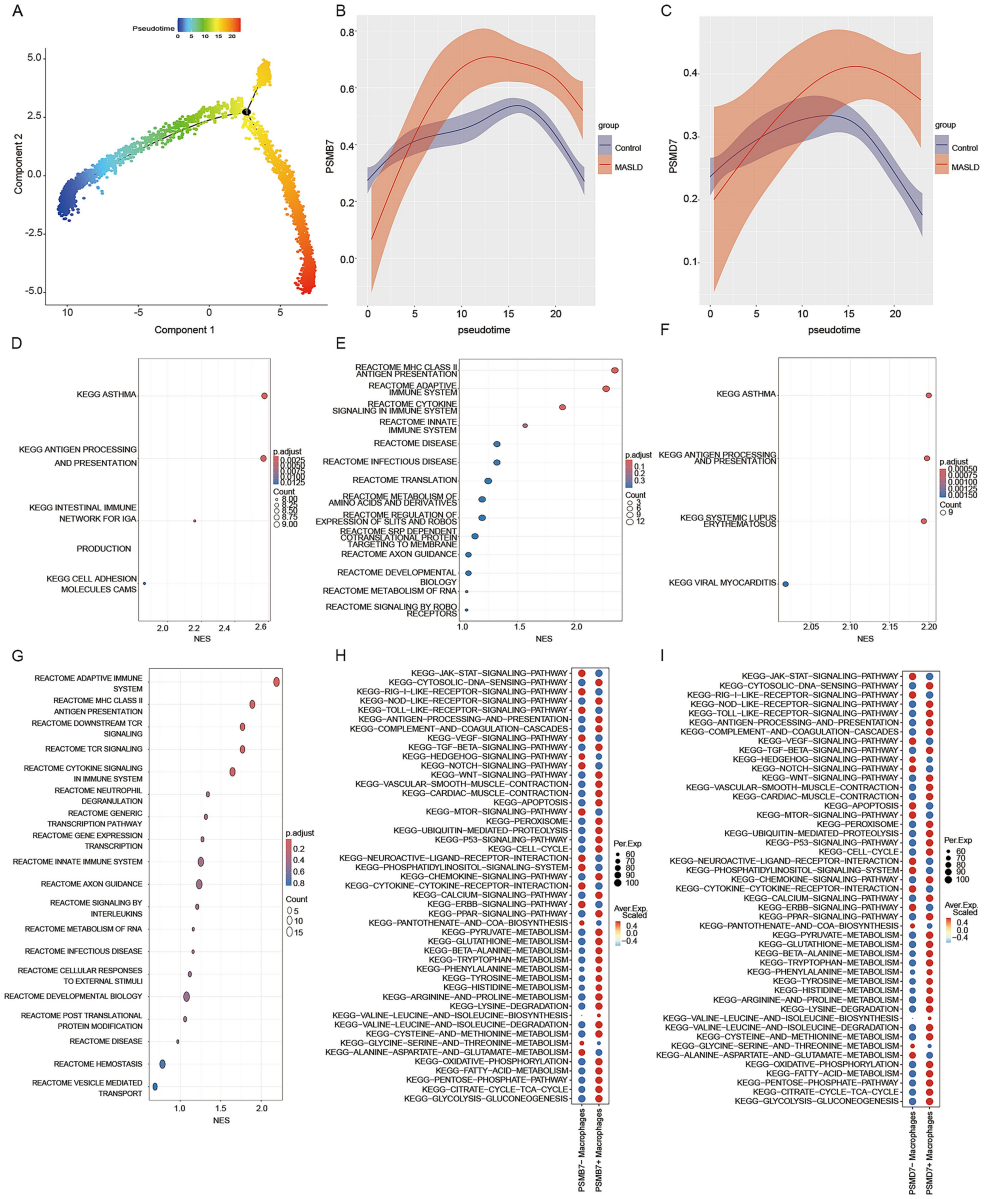
Pseudotime trajectory analysis and enrichment analysis. **(A)** Macrophage pseudotime trajectory. **(B,C)** Relative expression of PSMB7 and PSMD7 along pseudotime. **(D,E)** GSEA dotplot of KEGG/Reactome pathways enriched in PSMB7 + macrophage. **(F,G)** GSEA dotplot of KEGG/Reactome pathways enriched in PSMD7 + macrophage. **(H,I)** Dotplot of KEGG pathway activity scores (AUCell) in PSMB7 + and PSMD7 + macrophages.

Pseudotime trajectory analysis revealed that PSMB7 and PSMD7 expression increased along the macrophage trajectory in MASLD samples compared with controls, with peaks at intermediate states (Figures 8A–C). Functional enrichment analysis indicated that higher expression of these genes was associated with MHC class II antigen presentation, adaptive immune response, and cytokine signaling signatures (Figures 8D–G). Further comparison of macrophage subpopulations showed that cells with high PSMB7 or PSMD7 expression were enriched for multiple innate immune signaling pathways, including JAK–STAT, cytosolic DNA-sensing, and Toll-like/NOD-like/RIG-I-like receptor pathways (Figures 8H,I). These results suggest that elevated PSMB7/PSMD7 expression is associated with macrophage states enriched for inflammatory and antigen-presentation-related transcriptional programs.

### Validation of PSMB7 and PSMD7 upregulation in the CDAA-induced MASH model

**Figure 9:**
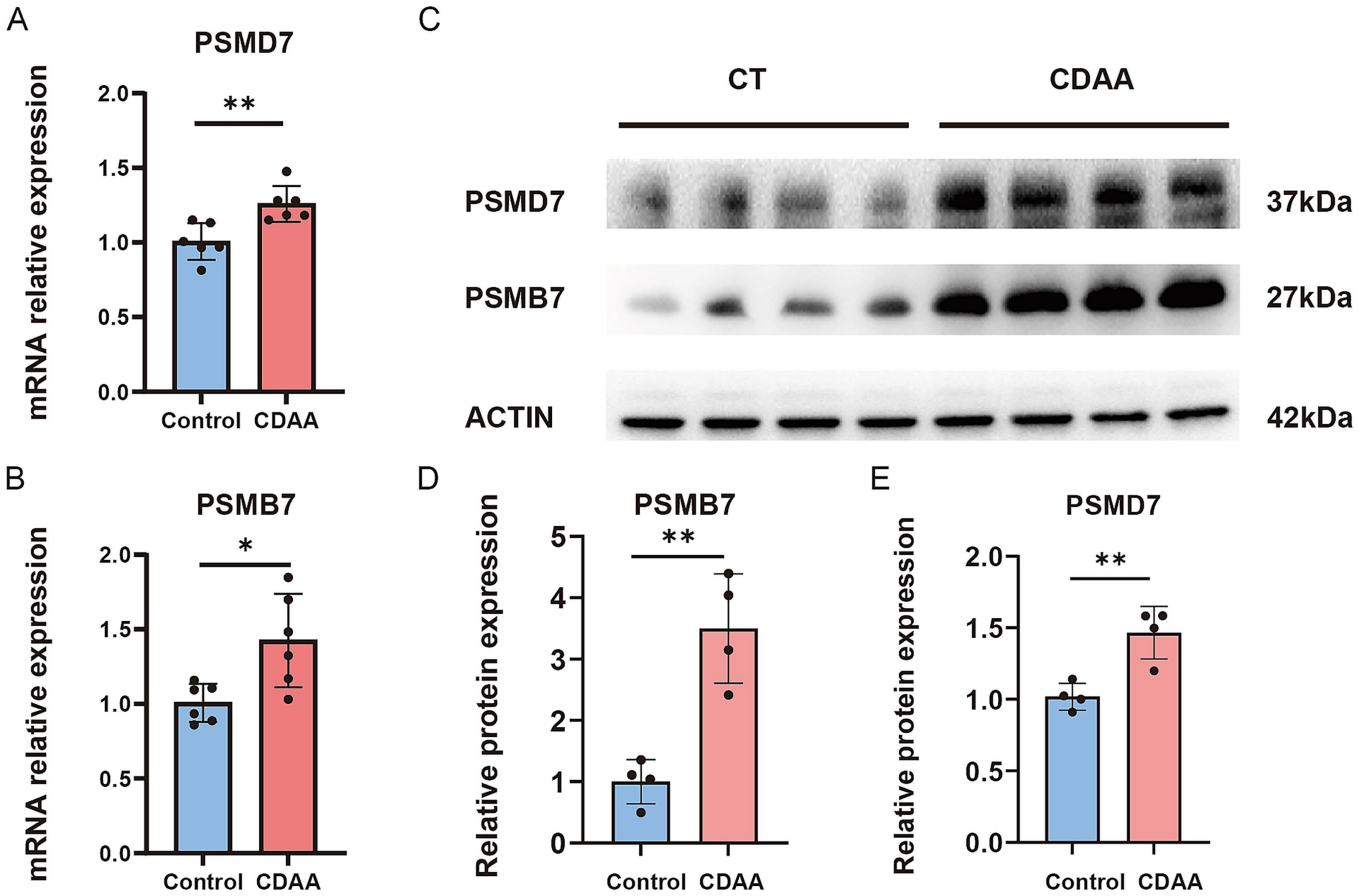
Validation of PSMB7 and PSMD7 expression in mouse MASH models. **(A,B)** The results of qRT-PCR showed that PSMD7 and PSMB7 significantly increased in MASH (metabolic dysfunction-associated steatohepatitis) model compared to control group (*n* = 6/group). **(C–E)** The results of Western Blot indicated that PSMD7 and PSMB7 increased in MASH model (*n* = 4/group). Data are presented as mean ± S.D. Two-tailed Student’s t-test for **A, B, D, E**; *p < 0.05, **p < 0.01. CDAA, choline-deficient, L-amino acid-defined.

To further support the transcriptomic findings, PSMB7 and PSMD7 were evaluated in a CDAA-induced MASH mouse model (Supplementary Figure S1). The mRNA expression levels of both PSMB7 and PSMD7 were significantly increased in the CDAA group compared to the control group (Figures 9A,B). Consistent with these findings, western blot analysis showed that the protein abundance of PSMB7 and PSMD7 was also elevated in CDAA-treated mice (Figures 9C–E). Together, these results support the transcriptomic observation that PSMB7 and PSMD7 are upregulated in MASH-related liver injury.

## Discussion

Metabolic dysfunction-related fatty steatotic disease, formerly known as non-alcoholic fatty liver disease, has now become a major global health problem, reflecting the rise of metabolic syndrome and obesity (3). The transformation from simple fatty change to metabolic dysfunction-related steatohepatitis is a key clinical turning point, as described in the recent Delphi naming consensus (1). The course of this disease is mainly driven by a combination of three factors: metabolic imbalance, lipotoxicity, and chronic low-grade inflammation (7). From a histological point of view, MASH shows the expansion of hepatocytes, Mallory-Denk bodies and the intensification of fibrosis. Fibrosis is the strongest factor in predicting long-term death (29, 30). Genetic factors such as PNPLA3 mutations increase the risk of disease (31), but metabolic pathways are still more important. Increasing attention has focused on the role of immune-metabolic interactions in this transition. Among the metabolic pathways involved, polyamine metabolism has emerged as a potential contributor to liver inflammation and metabolic imbalance (11, 32).

Spermidine, a naturally occurring polyamine, has been reported to exert protective effects in the liver (33). One important mechanism involves the hypusination of the translation factor eukaryotic initiation factor 5A (EIF5A), a unique post-translational modification catalyzed by deoxyhypusine synthase (DHPS) and deoxyhypusine hydroxylase (DOHH) (33, 34). This modification is essential for mitochondrial protein translation and the maintenance of mitochondrial fatty acid *β*-oxidation (FAO). Previous studies have shown that DOHH expression is markedly reduced in the liver of patients with metabolic dysfunction-associated steatohepatitis as well as in experimental mouse models, resulting in impaired EIF5A hypusination (33, 35). The resulting mitochondrial dysfunction can aggravate lipid toxicity and hepatocellular injury. In contrast, accumulation of upstream polyamines such as putrescine appears to promote inflammatory responses. In TH17 cells, the transcription factor HIVEP1 can transcriptionally activate ornithine decarboxylase 1 (ODC1), leading to metabolic rewiring of the polyamine pathway and excessive production of putrescine (11). Elevated putrescine levels have been linked to the acquisition of a pro-inflammatory TH17 phenotype and increased secretion of cytokines such as IL-17A (11). In addition, polyamines may influence the hepatic immune microenvironment by regulating macrophage polarization. Several studies suggest that polyamines can reshape macrophage transcriptional programs through epigenetic mechanisms, limiting their transition toward the reparative M2 phenotype while sustaining a pro-inflammatory M1-like state (36). Persistent inflammatory signaling from macrophages can subsequently activate hepatic stellate cells (HSCs) through paracrine pathways, thereby promoting extracellular matrix deposition and liver fibrosis (37). These observations have raised interest in targeting polyamine metabolism for MASLD therapy. For instance, spermidine supplementation has been proposed to restore mitochondrial metabolic homeostasis, whereas pharmacological inhibition of ODC1 using difluoromethylornithine (DFMO) may suppress TH17-driven inflammatory responses.

In this study, we combined single-cell RNA sequencing with multi-algorithm feature prioritization to construct a cell-type-resolved map of polyamine-metabolism-associated transcriptional heterogeneity in MASLD (38, 39). Rather than establishing a definitive mechanism, our analyses were designed to identify candidate genes and cellular states linked to polyamine-high transcriptional programs. Using this framework, we found that macrophages showed relatively high polyamine-metabolism scores and that the proteasome-associated genes PSMB7 and PSMD7 were repeatedly prioritized across multiple analytic layers. These observations extend the current literature by providing a macrophage-centered, single-cell view of polyamine-metabolism-associated remodeling in MASLD and by nominating candidate genes for future mechanistic investigation.

Notably, the proteasome system is functionally connected with polyamine metabolism through the antizyme regulatory pathway (40, 41). Previous studies have established important links between polyamine metabolism and immune-cell function, including validated mechanisms involving ODC1-related pathways in inflammatory macrophages and TH17 cells (42). We therefore interpret these genes as proteasome-associated candidate markers linked to macrophage populations with elevated polyamine-metabolism scores, rather than experimentally proven master regulators of polyamine metabolism.

Furthermore, the ubiquitin–proteasome system is also the main system of protein degradation and antigen processing in cells (43). In the MASLD liver, the increase in these subunits is likely to be a response to misfolded proteins and endoplasmic reticulum stress, both of which are typical features of lipotoxic liver damage (44). New research shows that proteasome dysfunction or overactivation can induce a variety of cell deaths in the liver, including apoptosis and ferroptosis (45, 46). Our analysis shows that the high expression of PSMB7 and PSMD7 is positively correlated with the infiltration of activated CD8 T cells and effect memory CD4 T cells, which are known to promote self-directed liver damage in MASH (47). This shows that the proteasome activity driven by these genes may increase the production of MHC I peptides, thus promoting abnormal autoimmune responses in the liver. Similar effects occur in other inflammatory environments, and the immune proteasome is activated. Therefore, PSMB7 and PSMD7 are not just passive damage signs. They actively participate in reshaping the immune environment. They help the liver change from a state of metabolic stress to a state of strong inflammatory signals and may act as a driving factor for the “second hit” in the MASH progression model (48).

Another key result of our research is that polyamine metabolism associated transcriptional states are unevenly distributed across hepatic cell types. We identified macrophages as the main cells with high polyamine metabolic activity, and also the cells with the strongest expression of PSMB7 and PSMD7. Liver macrophages are a hybrid class, including resident Kupffer cells and mononuclear-derived macrophages, which differ in origin and function (49). Trajectory analysis shows that with the progress of MASLD, macrophages change from a more homeostatic state to a highly inflammatory state with the ability to present strong antigens. This change is also described in other studies on liver macrophage diversity and fibrotic ecological positions (12). This metabolic reprogramming is usually driven by epigenetic and transcriptional changes, so that macrophages adapt to a nutrient-rich and inflammatory environment (12). Using CellChat and other intercellular communication tools, we further found that PSMB7 and PSMD7 macrophages build complex communication networks through MIF and LGALS9 pathways. MIF is a well-known cytokine that promotes chronic liver disease and attracts inflammatory cells (50). The interaction of LGALS9–CD44 is related to T cell depletion and immune control in the liver (51). By interacting with CD74 and CXCR4 receptors on B cells and endothelial cells, these “metabolically reprogrammed” macrophages create and maintain a pro-inflammatory site to support chronic inflammation. This indicates that PSMB7/PSMD7-high macrophages are associated with an inflammatory immune microenvironment in MASLD.

We acknowledge several limitations in this study, despite our integrative use of bioinformatics and machine learning. A key constraint is our heavy reliance on public transcriptomic data, which inherently differ in patient demographics and technical platforms. Broader validation across more diverse, prospective patient cohorts is essential to confirm how widely our findings apply. In addition, the scRNA-seq data used in this study were obtained from a public repository with a relatively small sample size, which precluded reliable stratified analysis or statistical adjustment for potential sex-related differences. We therefore refrained from drawing conclusions regarding sex-specific effects, and future studies with larger, balanced cohorts are needed to address this important question. On the mechanistic side, while we observed higher expression of PSMB7 and PSMD7 in the CDAA mouse model, we did not directly track polyamine metabolic flux. This means the exact link between these proteasome subunits and actual polyamine output is still not pinned down. Furthermore, our computational work points to these genes as potential drivers of immune cell recruitment, but this remains a correlative finding. Proving causation will require targeted functional studies, such as macrophage-specific knockout experiments. Even with these caveats, the consistent upregulation of PSMB7 and PSMD7 in macrophages across both multi-omics data and animal models firmly establishes their centrality in MASLD. This positions them as compelling candidates for targeting metabolic-immune crosstalk and lays a solid groundwork for future mechanistic and translational research.

## Conclusion

This study provides a single cell informed view of polyamine metabolism associated heterogeneity in MASLD and highlights macrophages as a major cell population linked to this remodeling. PSMB7 and PSMD7 emerged as proteasome-associated candidate genes enriched in macrophage populations with elevated polyamine-metabolism scores and associated with immune-related transcriptional features. These findings are hypothesis-generating and support future mechanistic, metabolomic, and functional studies to clarify how proteasome-related programs intersect with macrophage immunometabolism in MASLD.

## Data Availability

The original contributions presented in the study are included in the article/Supplementary material, further inquiries can be directed to the corresponding author.

## Footnotes

1
https://www.ncbi.nlm.nih.gov/geo

2
https://www.gsea-msigdb.org/gsea/msigdb

3
https://www.networkanalyst.ca/NetworkAnalyst
