# Supplementary material for: Integrating single-cell RNA-seq and machine learning to dissect polyamine metabolism in metabolic dysfunction-associated steatotic liver disease
Source: Front Med (Lausanne). 2026 Apr 29;13:1786869. doi: 10.3389/fmed.2026.1786869 (PMC13167413; doi:10.3389/fmed.2026.1786869)
Supplement: Supplementary file 1 [file Data_Sheet_1.PDF]

Fig. S1

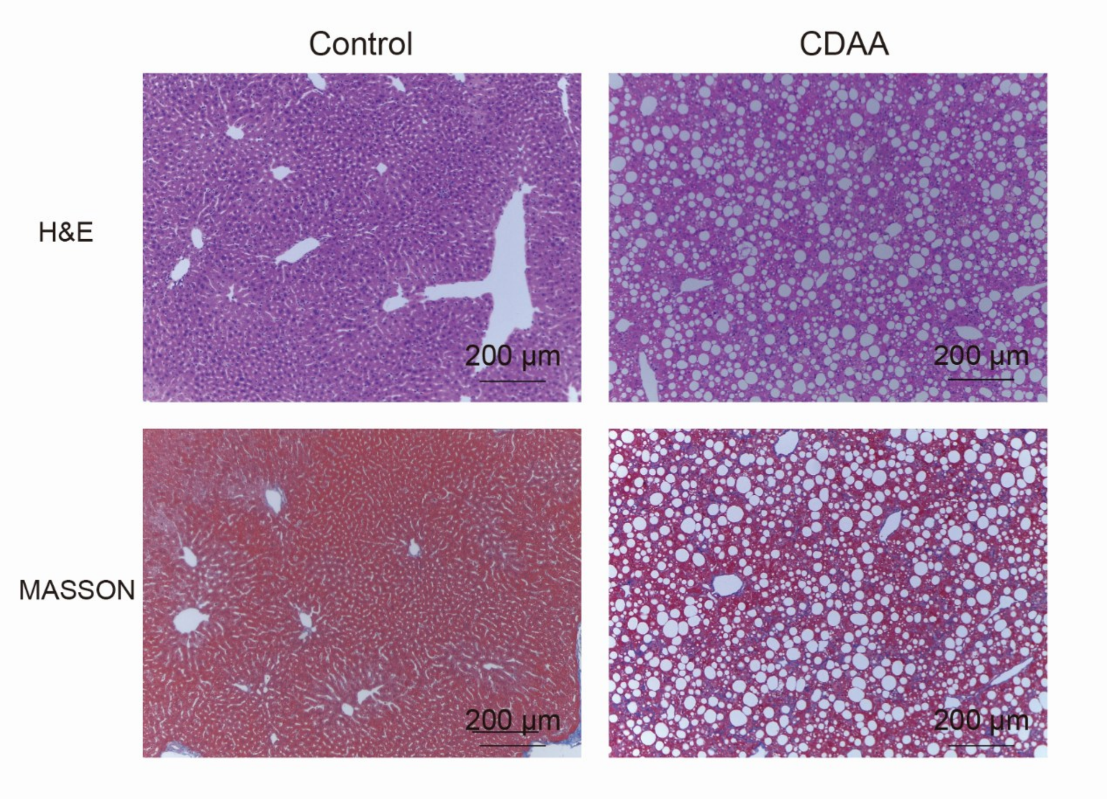

Liver histopathological sections stained with H&E (Hematoxylin and Eosin staining) and Masson from control mice and MASH mice.
